# Effect of a generalist mesopredator on modular and unitary sessile prey associated with a foundation species

**DOI:** 10.1002/ece3.11413

**Published:** 2024-05-15

**Authors:** Alexandra Chava, Anna Artemieva, Eugeniy Yakovis

**Affiliations:** ^1^ Laboratory of Ecology of Coastal Benthic Communities P.P. Shirshov Institute of Oceanology RAS Moscow Russia; ^2^ Department of Invertebrate Zoology St.‐Petersburg State University Saint‐Petersburg Russia

**Keywords:** bryozoans, community ecology, consumer control, epibiosis, modular, predation, serpulids, shrimp, unitary

## Abstract

Unitary and modular sessile organisms both dominate in marine benthic communities, commonly preyed upon by the same generalist predators. The differences between unitary and modular defensive strategies may underlie the ways generalist predators control community structure, but this has never been empirically examined. We hypothesize that the individual size of an omnivorous mesopredatory shrimp affects the relative vulnerability of unitary and modular prey and hence translates into community structure. In a short‐term laboratory microcosm experiment, we assessed the effect of the shrimp individual size on an epibiotic assemblage of red algae blades initially dominated by three species of modular bryozoans and a unitary serpulid tubeworm. We found that the individual size of a shrimp determines its effect on the prey community composition. Large shrimp stronger than small shrimp increased the proportion of unitary tubeworms among the epibionts surviving predation. While large shrimp reduced the proportions of all the three dominant bryozoan species, small shrimp, in contrast, mostly increased the proportion of a bryozoan species with the smallest modules and largest colonies. This bryozoan, like the tubeworms, demonstrated a higher survival rate with larger individual (colony) size. Yet, against large shrimp this bryozoan was outperformed by the largest tubeworms almost immune to predation. Partial predation by small shrimp modestly improved survival of the largest bryozoan colonies. Thus, relative vulnerability of unitary and modular prey is determined by the predator individual size. Our findings clarify the complex way the size structures of generalist consumers and their prey shape communities by affecting the species‐specific relative performance of modular and unitary organisms. The demography of a foundation species and the competitive hierarchy can have additional effects by altering the balance of predation and competition.

## Graphical abstract

In a short‐term laboratory microcosm experiment, we assessed the effect of the mesopredator shrimp individual size on an epibenthic assemblage of red algae initially dominated by three species of modular bryozoans and a unitary serpulid tubeworm. Our findings elucidate the complex way the size structures of generalist consumers and their prey shape communities by affecting the species‐specific relative performance of modular and unitary organisms. The demography of an algal foundation species and the competitive hierarchy of epibionts can have additional effects by tuning the balance of predation and competition.

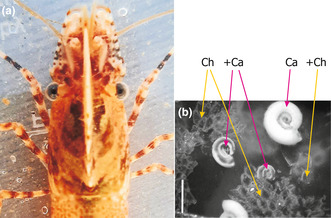

## 1 INTRODUCTION

Multicellular organisms feature either unitary (solitary) or modular (clonal, colonial) design. Modular vascular plants, algae, corals, hydroids, and bryozoans iterate genetically identical structural units as they grow, while unitary vertebrates, arthropods, bivalves, and worms grow by increasing the size of a single individual body. Unlike terrestrial communities, which are mainly composed of immobile modular and mobile unitary taxa, marine benthic communities host unitary and modular organisms that are both sessile. When acting as foundation species, both types provide habitat structure to multiple dependent taxa and thus define entire landscapes, for instance, coral reefs or bivalve beds. Frequently, immobile unitary and modular organisms share common habitats and codominate the same communities. Numerous examples include co‐dwelling giant clams and corals (Hamner & Jones, 1976; Hardy & Hardy, 1969; Lucas, 1988; Soo & Todd, 2014), and bivalves, tubeworms, ascidians, sponges, hydroids, and bryozoans in various combinations (Barnes & Clarke, 1995; Chava et al., 2019; Davis & White, 1994; Hiebert et al., 2019; Nandakumar et al., 1993; Velimirov et al., 1977). Coexisting immobile unitary and modular organisms commonly compete for substrate space (Jackson, 1977).

Consumer control (predation and grazing) is one of the primary processes in community regulation. Unitary and modular organisms mitigate the pressure of consumers differently. Modular organisms replicate their vital systems within modules across a clone. As a result, while consumer attacks are commonly fatal for unitary organisms, modular ones can survive partial damage by sacrificing some of the modules (Dyrynda, 1986). Clones are able to grow into refuges and later repair the lost parts; therefore, larger clones have better chances to save enough modules to survive. On the other hand, predation or grazing on modular prey can selectively target specific module types, for example, the most nutrient‐rich reproductive ones, indirectly affecting recruitment in addition to survival (Sallabanks & Courtney, 1992). Unitary prey is rarely subject to partial predation, but commonly manages to outgrow consumer pressure by increasing their bodies along with defensive structures beyond a safety threshold called “escape size”. Giant clams and mussels, for example, become increasingly safe from predation as they grow (Paine et al., 1985; Soo & Todd, 2014; Waters et al., 2013). In addition, as fertility often comes with size, breeding unitary individuals are relatively large, which makes them less vulnerable. Unitary and modular organisms are commonly consumed by the same generalist predators or grazers. For instance, many common species of reef pufferfishes and triggerfishes consume both juvenile giant clams and corals (Cole et al., 2008; Neo et al., 2015), and Atlantic purple sea urchins graze on ascidians, bryozoans, sponges, barnacles, and oysters (Karlson, 1978).

Defensive strategies employed by modular and unitary organisms have been extensively studied separately (e.g., Beukema & Dekker, 2005; Dyrynda, 1986; Hiddink et al., 2002), but to our knowledge, there is only scarce empirical data on their comparative efficiency against a particular omnivorous predator or grazer in a multispecies community (Karlson, 1978). We suggest that relative performances of outgrowing predation pressure (more expected in unitary prey) and sacrificing part of the modules by modular prey would change with the individual size of a predator, constituting an important mechanism of community regulation. Exploring the relationship could reveal the way unitary and modular defensive strategies translate consumers' population size structure into prey community structure. To date, few controversial field caging experiments, where all the predators larger than mesh cell size were excluded, provide almost opposite results depending on the system studied. Total predator removals favor solitary tubeworms and ascidians against colonial ascidians (Mook, 1983), colonial ascidians against solitary ones (Hiebert et al., 2019), and recruits of colonial bryozoans against solitary tubeworms and cirripeds (Sowa et al., 2023). Other field experiments do not show advantages in tolerating consumer control distinctly associated with unitary or modular organization (Osman et al., 1992; Sams & Keough, 2007; Vieira et al., 2012). In the study by Hiebert et al. (2019), colonial ascidians still dominate the unmanipulated community, apparently since they regrow when partially consumed and adjust in shape and space to grow into refuges. The contradictory outcomes of these experiments are seemingly caused by the interplay of selective consumer control and prey recruitment and growth. At the same time, the relative importance of a specific mechanism and the roles of particular predators (probably having species‐ and size/age‐specific diets) remain unclear.

Here, we assessed the effect of consumer control in short‐term microcosm experiments. We exposed a natural epibiosis of red algae blades dominated by unitary and modular sessile organisms to a common generalist mesopredator (shrimp) to isolate predation from other processes. The time scale selected was short enough to avoid any growth and propagation of the potential prey and non‐consumptive predator effects (Johnson & Strathmann, 1989). Omnivorous predators often exhibit diet shifts with size (Sánchez‐Hernández et al., 2018). Predator size can also affect the escape size of the prey (Kelley & Hunsen, 1996). We hypothesized that this would result in the difference in community composition changes caused by small and large predator individuals. We expected the ability of unitary species to outgrow predation pressure and the ability of modular ones to be consumed partially would change with predator size, providing the link between the demography of the predator and community response to predation. Importantly, the focal system is shaped by the demography of a foundation species (Chava et al., 2019), which provides the background to examine the complex interplay of predation, facilitation, and competition driving community structure and functioning.

## 2 MATERIALS AND METHODS

**FIGURE 1:**
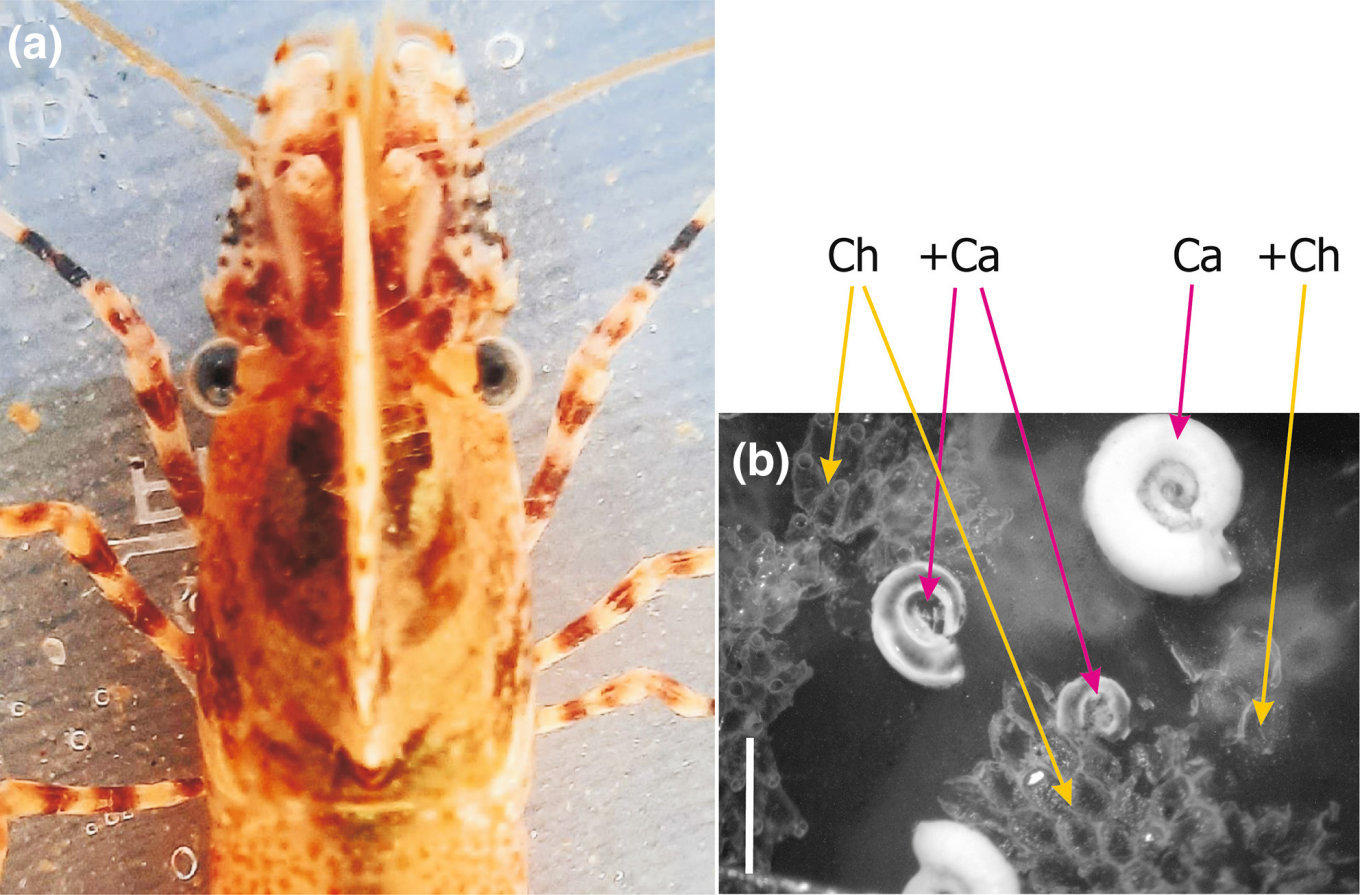
*Spirontocaris phippsii* head and thorax, top view (a), and a sample fragment of *Phycodrys rubens* epibiosis after a 24‐h trial with *S. phippsii* (b). Scale bar is 1 mm. “Ca”—live *Circeis armoricana*, “+Ca”—remains of *C. armoricana* destroyed by *S. phippsii*, “Ch”—live zooids of *Celleporella hyalina*, “+Ch”—remains of *C. hyalina* zooids destroyed by *S. phippsii*.

Young blades of a red foliose algae *Phycodrys rubens* in the White Sea host an epibiosis codominated by unitary serpulid tubeworms *Circeis armoricana* and modular encrusting bryozoans *Celleporella hyalina*, *Juxtacribrilina annulata*, and *Electra pilosa* (Chava et al., 2019). Punctate blade shrimp *Spirontocaris phippsii* (Figure 1a) is a common generalist mesopredator previously known to prey on small polychaetes, amphipods, bivalves, and gastropods (Yakovis & Artemieva, 2019, 2021).

To assess the effect of *Spirontocaris phippsii* (hereafter *Spirontocaris*) predation on epibiotic assemblages, we collected random young (less than a year old) *Phycodrys rubens* blades covered with epibiosis in Velikaya Salma Strait (Kandalaksha Bay of the White Sea) at 66°33.276′ N, 33°6.470′ E on August 4, 13, and 29, 2014. Young blades emerge in May, make up the most part of *P. rubens* thallus and can be easily distinguished from the older part of the plant (Schoschina, 1996). For each trial, we exposed a new single randomly chosen blade (mean total area of both sides: 1052 ± 59 mm^2^) in a 300 mL aerated tank at 9–10°С for 24 h, stem anchored to the tank bottom. All sessile macrobenthic organisms on a blade were counted, and individually measured (zooids number in bryozoans and hydrozoans, aperture diameter in tubeworms) in the beginning and at the end of each trial. Substrate area covered was estimated using previously established relationships (Chava et al., 2019). We performed 24‐h experimental trials with a single *Spirontocaris* individual added to a tank (Figure 1b). We used 18 shrimp individuals each utilized in three trials with one exception when a shrimp was utilized in two trials. In total, there were 53 trials. Shrimp were acclimated to tank conditions and starved for several days before trials.

Growth and mortality rates of epibionts were low enough to ensure the absence of traceable changes to sessile assemblages on blades within 24 h without predators. This was checked on nine additional randomly selected *Phycodrys* blades assigned to technical control trials which shared setup with experimental trials except we added no shrimp to the tanks. All sessile epibionts in these control trials were also counted and measured before and after exposure. Both numbers and sizes were identical after 24 h. Experimental and control trials had no significant difference (Student's *t*‐test) neither in mean average substrate area (*p* = .363) nor in initial total percent cover of the epibionts (*p* = .717).

To test the effect of predator size on community structure changes in the experimental trials we used the shrimp from the two contrasting size groups randomly assigned to treatments: “large” with six individuals each weighing 0.300–0.410 g (18 trials, hereafter “large shrimp trials” or “LST”) and “small” with 12 individuals each weighing 0.010–0.080 g (35 trials, hereafter “small shrimp trials” or “SST”).

The brief starvation before the trials could potentially reduce feeding selectivity in shrimp. Therefore, we controlled for this bias by testing the effects of the sequential order number of a replicate trial on (i) percent covers cleared by predators and (ii) species diversity of the prey consumed, with Friedman tests (separate for SST and LST).

Effects of predator size on the changes that predation caused to (i) mean total area covered, (ii) Shannon–Wiener species diversity, and (iii) species numbers were explored with Type III sum of squares GLM ANCOVAs. The pairwise differences in mean total area covered, Shannon–Wiener species diversity, and species numbers before and after each treatment were the response variables. Initial total percent cover and relative covers of each top dominant taxa (a tubeworm and three bryozoans) were the covariates, while shrimp size (large or small, fixed) and shrimp individual ID (random, nested in shrimp size) were the categorical predictors. Variances were homogenous (Cohran's test). We assessed the effect of predation on multivariate community composition by applying permutational analysis of variances (PERMANOVA, Anderson, 2017) with time (before or after treatments), shrimp size (large or small), and their interaction as fixed effects, and trial and shrimp IDs as random nested effects, followed by pairwise tests between the LST and SST before and after treatments. The analysis was performed on Bray–Curtis similarities calculated from standardized percent covers of all the epibenthic species identified. The differences were visualized in a non‐metric multidimensional scaling plot.

To assess the effect of partial predation, we compared mortality rate (decrease in the number of individuals/colonies) with loss of substrate area covered in the same trials. Shifts in size structure of the prey were assessed by comparisons of mean sizes of the prey before and after the treatments. We used the exact Wilcoxon–Pratt signed‐rank test (Hothorn et al., 2008) for these pairwise comparisons.

To examine the relationships between demography and survival in unitary versus modular prey, we assessed the effects of predator and prey size on prey mortality. Unitary tubeworms (the size of which remained constant throughout the experiments) allowed using individual size as a predictor of mortality, while for modular bryozoans (some of which were shrunk by partial predation), we used initial mean colony size in a trial as a predictor. We applied multiple zero‐ and one‐ inflated beta‐regression (Stasinopoulos & Rigby, 2008) to evaluate the effects of predator size (small or large) and initial mean colony size in a trial (for bryozoans) or individual size (for tubeworms), and also absolute and relative species abundances on the mortality. Individual shrimp ID (in all species) and trial ID nested in shrimp ID (in tubeworms only) were used as random blocking factors. Shrimp size was used as a predictor to model mortality variance (sigma) to account for the difference in variances between SST and LST. Calculations were performed using R version 4.1.3. Means are reported ±SE.

## 3 RESULTS

### 3.1 Species composition, diversity, and dominance

Large shrimp reduced total area covered by epibionts, species diversity, and species number more than small shrimp (Tables 1 and 2). There was no statistical difference between consequent replicate trials neither in Shannon–Wiener species diversity index of the consumed taxa (SST: *p* = .529, LST: *p* = .607, Friedman test) nor in the proportion of prey consumed in terms of total percent cover reduction (SST: *p* = .761, LST: *p* = .115, Friedman test).

**TABLE 1:** Results of ANCOVA on the differences in total substrate area covered by epibionts, their Shannon–Wiener diversity indexes, and species numbers before and after exposure to *Spirontocaris phippsii* predator shrimp in the laboratory experiment.

Source of variation		df	Difference in total area covered			Difference in species diversity			Difference in species number		
			SS	*F*	*p*	SS	*F*	*p*	SS	*F*	*p*
Intercept	Fixed	1	28.3	0.28	.603	0.051	0.70	.408	0.882	1.09	.305
Shrimp size [large, small]	Fixed	1	1236.7	8.83	.008**	1.283	12.51	.002**	24.879	14.96	.001**
Shrimp ID (Shrimp size)	Random	16	2274.8	1.39	.214	1.668	1.45	.183	27.352	2.11	.037*
Initial % of *Circeis armoricana*	Fixed	1	2.1	0.02	.888	0.004	0.06	.811	0.170	0.21	.650
Initial % of *Celleporella hyalina*	Fixed	1	3.9	0.04	.846	0.010	0.14	.713	0.378	0.47	.500
Initial % of *Juxtacribrilina annulata*	Fixed	1	26.6	0.26	.615	0.001	0.01	.923	0.002	0.00	.959
Initial % of *Electra pilosa*	Fixed	1	151.3	1.48	.234	0.001	0.02	.889	0.598	0.74	.397
Initial total area covered	Fixed	1	253.6	2.47	.126	0.160	2.24	.145	0.003	0.00	.956
Error		30	3077.1			2.151			24.269		

*Note*: *p < .05, **p < .01.

**TABLE 2:** Mean total area covered by epibionts, their percent cover, species diversity, and species number before and after exposure to *Spirontocaris phippsii* predatory shrimp in the laboratory experiment.

Time	Before^a^	After	
Shrimp size		Small	Large
Mean total area covered (mm^2^)	45.9 ± 4.2	27.7 ± 5.1	17.1 ± 5.0
Mean total percent cover (%)	5.0 ± 0.5	2.9 ± 0.5	1.6 ± 0.5
Mean Shannon–Wiener species diversity index	1.27 ± 0.02	0.97 ± 0.01	0.61 ± 0.02
Mean species number	5.68 ± 0.11	4.54 ± 0.04	2.83 ± 0.07

^a^ Before the experiments, there was no significant difference (Student's *t*‐test) between SST and LST in total area covered (*p* = .816), initial total percent cover (*p* = .250), species diversity (*p* = .703), and species number (*p* = .789).

The species composition of initial assemblages included 11 species: Eight bryozoans, two hydroids, and one serpulid tubeworm. Four top abundant species accounted for 95 ± 1% of total cover before the trials: a unitary serpulid tubeworm *Circeis armoricana* (hereafter *Circeis*) contributed 28 ± 2%, while modular cheilostome encrusting bryozoans *Celleporella hyalinа* (hereafter *Celleporella*), *Juxtacribrilina annulata* (hereafter *Juxtacribrillina*), and *Electra pilosa* (hereafter *Electra*) contributed 34 ± 3%, 22 ± 2%, and 11 ± 1%, correspondingly. The same four species also contributed 95 ± 1% to cover reduction in predator trials. Most trials included all the four dominants (with *Electra* initially present in 94%, *Celleporella* in 96%, *Circeis* and *Juxtacribrilina* in 100% of the trials).

**FIGURE 2:**
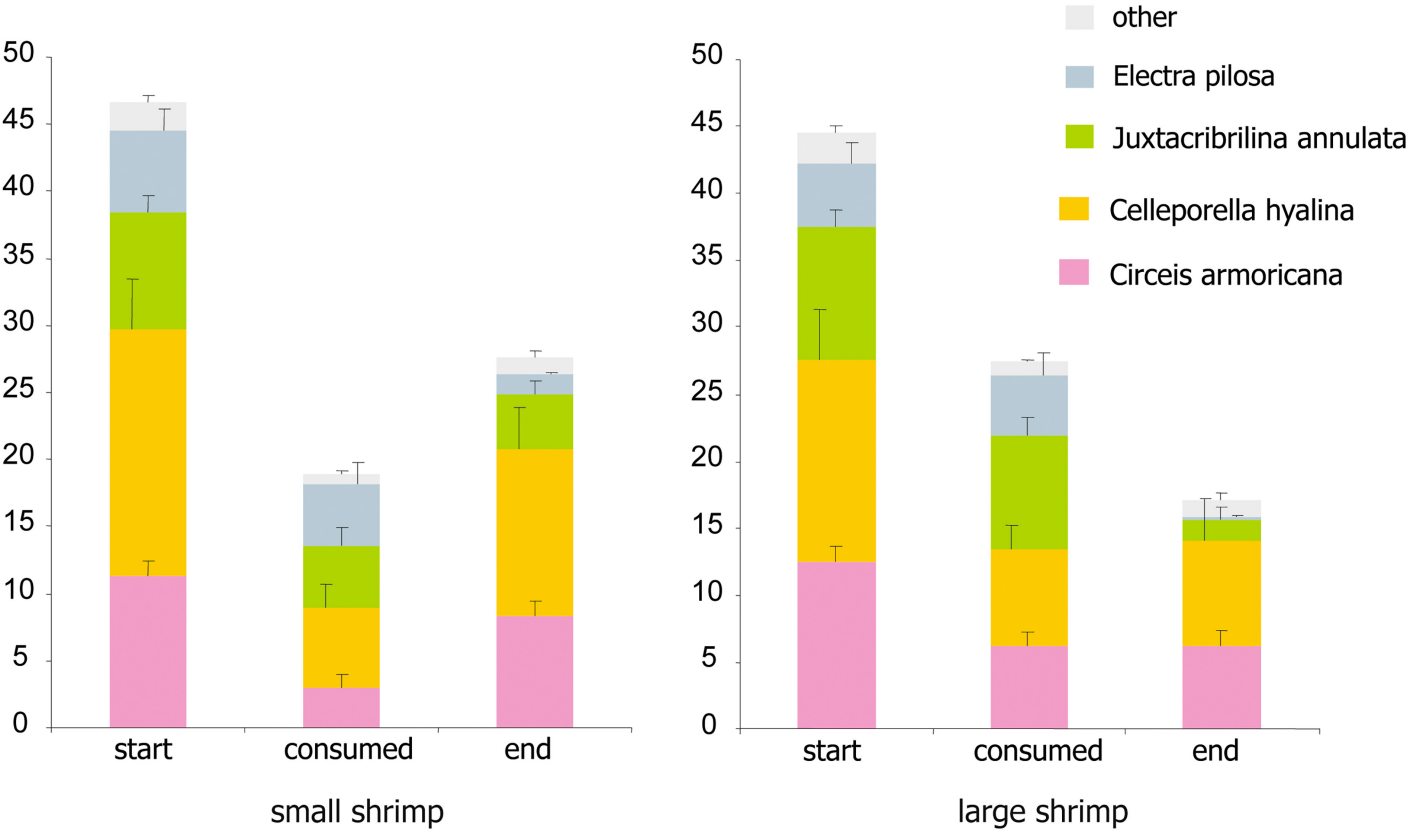
Mean area (mm^2^) occupied by dominant epibenthic taxa on *Phycodrys rubens* blades before and after exposure to *Spirontocaris phippsii* predatory shrimp in the laboratory experiments. “Start”—initial assemblages; “End”—surviving assemblages; “Consumed”—consumed by shrimp.

While shrimp profoundly reduced percent covers in all the dominants, large and small shrimp unequally consumed different prey species (Figure 2). Large shrimp caused a significantly higher increase in the proportion of unitary tubeworms (*Circeis*) and a significantly higher decrease in the proportions of modular *Juxtacribrilina* and *Electra* compared to small shrimp. The mean proportion of modular *Celleporella* was insignificantly decreased by large shrimp and increased by small shrimp (Table 3).

**TABLE 3:** Changes to relative abundances of dominant prey species and their elimination frequency after exposure to *Spirontocaris phippsii* predatory shrimp in the laboratory experiment.

Prey species		Circeis armoricana			Juxtacribrilina annulata			Celleporella hyalina			Electra pilosa		
Shrimp size		Small		Large	Small		Large	Small		Large	Small		Large
Mean relative abundance change (AC), %^a^		**+58 ± 14**	<	**+95 ± 20**	**−39 ± 9**	<	**−77 ± 9**	+2 ± 9		−14 ± 24	**−67 ± 7**	<	**−94 ± 6**
Mann–Whitney *U*‐test comparing AC between small and large shrimp trials	U	2.3			2.6			−1.3			2.5		
	*p*	.024*			.008**			.214			.011*		
The proportion of trials where the relative abundance of the species decreased (TD), %^b^		**14 ± 2**		**11 ± 2**	**77 ± 3**		**89 ± 2**	31 ± 4	<	61 ± 6	**86 ± 2**		**89 ± 2**
*χ* ^2^ test comparing TD between small and large shrimp trials	*χ* ^2^	0.10			1.07			4.84			0.16		
	*p*	.747			.301			.028*			.692		
The proportion of trials where the species was totally eliminated (TE), %^c^		0 ± 0		0 ± 0	**20 ± 7**	<	**50 ± 12**	6 ± 4	<	**47 ± 12**	**45 ± 9**	<	**88 ± 8**
*χ* ^2^ test comparing TE between small and large shrimp trials	*χ* ^2^	–			5.08			12.19			8.56		
	*p*	–			.024*			.005**			.003**		

*Note*: “<” sign indicates the significant difference in the parameter between small and large shrimp trials.

^a^ Highlighting in bold indicates significant effect of the trials on mean relative cover of the species (exact Wilcoxon–Pratt signed‐rank test, *p* < .001).

^b^ Highlighting in bold indicates significant deviation from 0.5 of the proportion of the trials where the species' relative abundance decreased (exact binomial test, *p* < .01).

^c^ Highlighting in bold indicates significant deviation from zero of the proportion of the trials where the species was totally eliminated (Chi‐square test, *p* < .01).

*p < .05, **p < .01.

**FIGURE 3:**
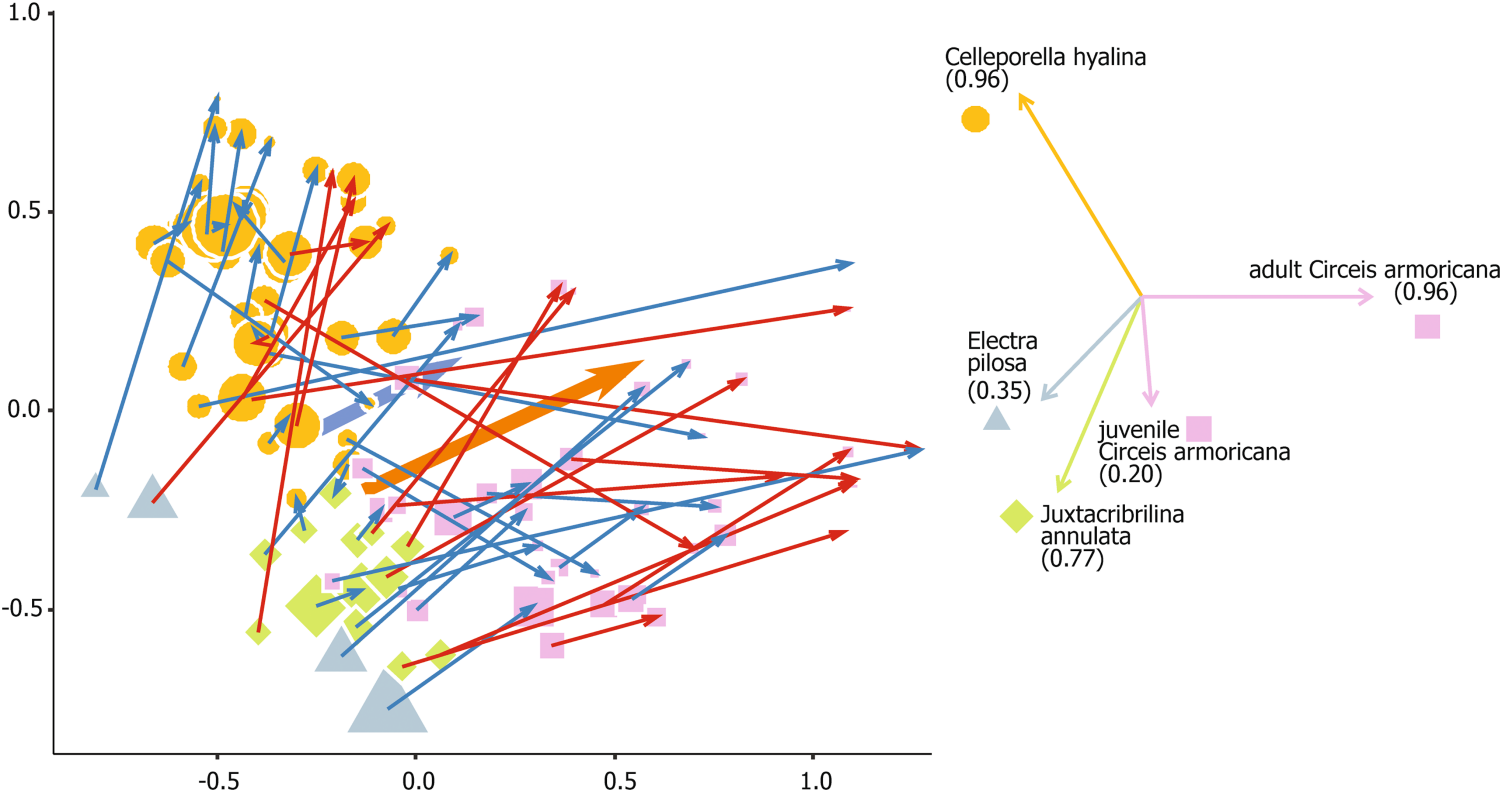
Non‐metric multidimensional scaling (nMDS) of the epibenthic assemblages on *Phycodrys rubens* blades before (arrow starts) and after (arrow ends) *Spirontocaris phippsii* predator shrimp exposure in the laboratory experiments. Blue arrows denote small shrimp trials, and red arrows denote large shrimp trials. Point marks indicate the dominant species. Point‐mark sizes denote the total substrate area covered. Large arrows connect centroids calculated for small (blue) and large (red) shrimp trials. Bray–Curtis similarity on standardized percent covers. Dominant species covers' relationships with ordination axes plotted with *R* ^2^ (in brackets); juvenile (≤0.15 mm aperture diameter) *Circeis armoricana* shown separately.

Multivariate analysis (Table 4) showed that initially similar species compositions were differently altered in SST and LST. Non‐metric MDS ordination (Figure 3) indicated that small shrimp had a weaker effect, while the direction of changes in species relative abundances was collinear for LST and SST. *Circeis* increased its relative cover in most LST and SST, and *Juxtacribrilina* and *Electra* decreased their relative cover in most LST and SST. In contrast, the proportion of outcomes for *Celleporella* was significantly affected by shrimp size: large shrimp mostly reduced the relative cover of *Celleporella*, while small shrimp mostly increased the relative cover of *Celleporella* (Table 3).

**TABLE 4:** Results of PERMANOVA (multivariate analysis of variance) on standardized substrate area covered by epibionts (Bray–Curtis dissimilarities) before and after exposure to *Spirontocaris phippsii* predatory shrimp in the laboratory experiment.

Source of variation		df	SS	MS	Pseudo‐*F*	*p*	Unique permutations
Time [before, after]	Fixed	1	16,650	16,650	45.8	.0001***	9953
Shrimp size [large, small]	Fixed	1	5551	5551	3.1	.0680	9949
Shrimp ID (Shrimp size)	Random	16	28,393	1775	1.2	.2473	9898
Time × Shrimp size	Fixed	1	1854	1854	5.1	.0064**	9958
Trial ID (Shrimp ID (Shrimp size))	Random	35	50,864	1453	4.0	.0001***	9859
Error		51	18,549	364			

Pairwise tests, term Time × Shrimp size for pairs of levels of Shrimp size [large, small] within the levels of Time			
Level of time	*t*	*p*	Unique permutations
Before	1.36	.1530	9952
After	1.88	.0425*	9948

*Note*: *p < .05, **p < .01, ***p < .001.

**FIGURE 4:**
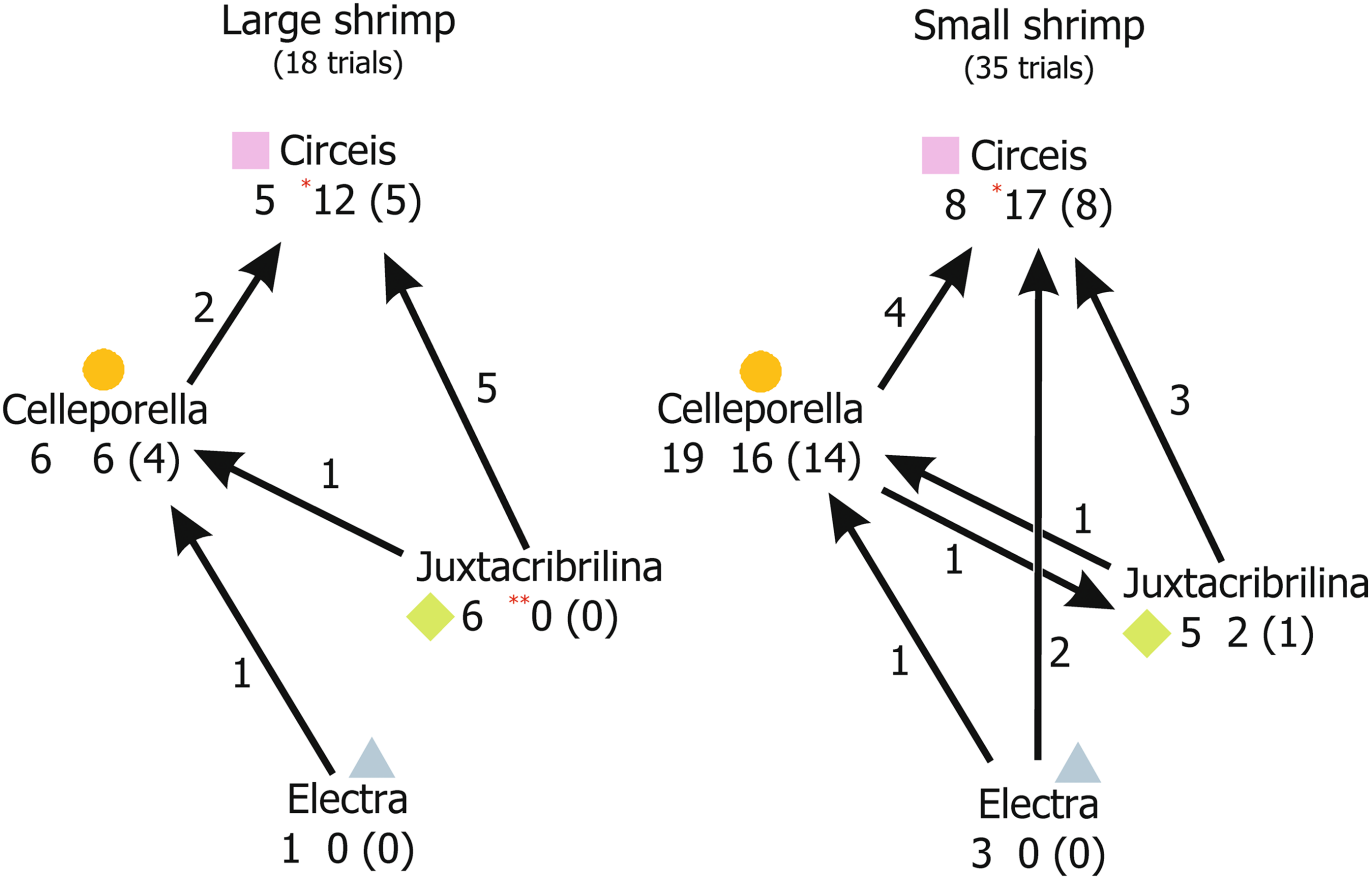
Shifts of domination resulting from exposure of the epibenthic assemblages on *Phycodrys rubens* blades to *Spirontocaris phippsii* shrimp predation in the laboratory experiments. Large arrows show the domination transitions with their counts. Numbers and small arrows under the taxa names denote the changes in domination frequency of the particular species, that is, “number of the trials initially dominated by a species” ↓ “number of the trials dominated by a species after shrimp exposure” (“number of the trials where domination of a species was not altered”). Small arrows with asterisks denote significant changes in domination frequency (Chi‐square, *p < .05, **p < .01).

Small and large shrimp both shifted dominance from *Juxtacribrilina* and *Electra* to *Circeis*. Moreover, *Juxtacribrilina* and *Electra* almost completely lost dominance to *Celleporella*. *Celleporella* sometimes lost to *Circeis*, while *Circeis* only gained dominance and never lost it (Figures 3 and 4). *Circeis* was the only species neither small nor large shrimp ever completely eliminated in any trial, while the other three were eventually eliminated by both. Small shrimp reduced the presence frequency of *Juxtacribrilina* and *Electra*, while large shrimp also reduced the presence frequency of *Celleporella* (Table 3).

### 3.2 Changes to size structure: Partial damage and size‐dependent survival

The overall contribution of partial predation to modular prey survival (as inferred from mean disparity between mortality and loss in substrate area covered) was small and limited to *Celleporella* in SST (Table 5). In *Juxtacribrilina*, mortality was only slightly lower than cover loss, significantly in LST and insignificantly in SST. There were no differences between mortality and cover loss in *Electra*. *Celleporella*, however, had 26% lower mortality than cover loss in SST. Both large and small shrimp reduced the number of *Circeis* individuals more than substrate area covered (Table 5). Yet, the largest bryozoan colonies generally survived better than average ones (see below).

**TABLE 5:** Mean mortality (%) and percent cover loss (%) in dominant prey species exposed to *Spirontocaris phippsii* predatory shrimp in the laboratory experiment.

Shrimp size	*Circeis armoricana*			*Juxtacribrilina annulata*			*Celleporella hyalina*			*Electra pilosa*	
	Mortality		Cover loss	Mortality		Cover loss	Mortality		Cover loss	Mortality	Cover loss
Small	**40 ± 4**	>	**33 ± 4**	59 ± 5		61 ± 6	**34 ± 6**	<	**43 ± 6**	77 ± 5	79 ± 5
Large	**60 ± 7**	>	**51 ± 7**	**85 ± 6**	<	**88 ± 6**	72 ± 9		68 ± 9	94 ± 6	94 ± 6

*Note*: Significant differences (exact Wilcoxon–Pratt signed‐rank test, *p* < .05) between mortality and cover loss highlighted in bold, “<” – cover loss higher than mortality, “>” – mortality higher than cover loss.

**FIGURE 5:**
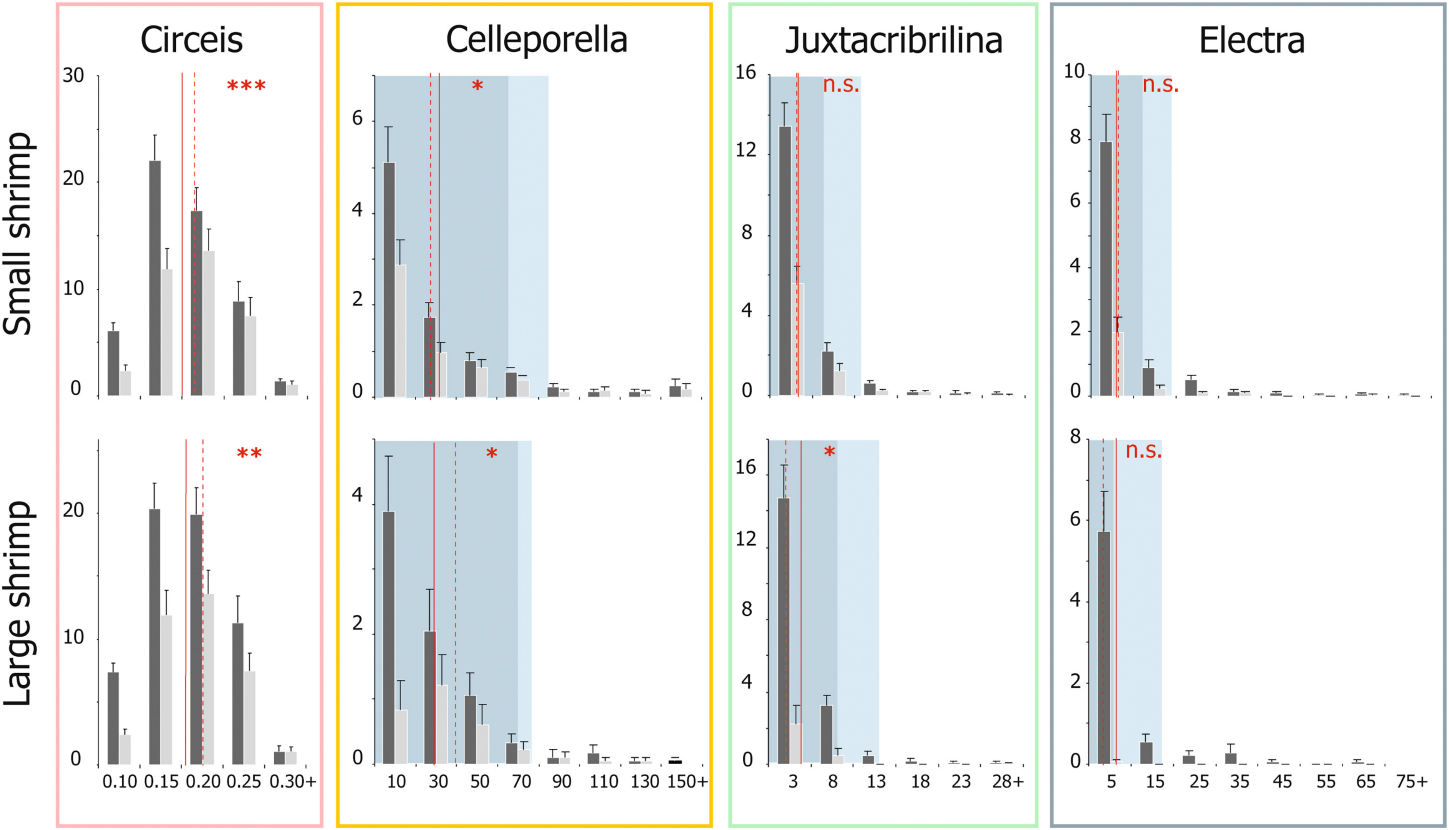
Size structures of dominant epibenthic species on *Phycodrys rubens* blades before (black bars) and after (gray bars) the exposure to *Spirontocaris phippsii* shrimp predation in the laboratory experiment. Red lines and arrows denote the changes in mean individual size (solid lines—before treatments, dashed lines—after treatments, *p < .05, **p < .01, ***p < .001, n.s.—not significant; exact Wilcoxon–Pratt signed‐rank test, see Table 5 for details). Blue bars show the mean largest colony size for bryozoans, the lighter bar denotes the size before; and the darker bar—after the treatments.

According to size structure changes, survival was generally size‐dependent in unitary and some modular prey, but the net species‐specific effect depended on the strength of the size–mortality relationship, predator size, and initial prey size structure (Table 6, Figure 5). Initially, the smallest colonies were the most frequent size class in bryozoans, while medium‐sized individuals prevailed in *Circeis*. *Celleporella* had both the smallest (due to a tiny zooid size) and the largest colonies. Both small and large shrimp significantly increased the mean individual size in *Circeis*, indicating selective size‐dependent predation pressure. While there was no statistical difference in the initial *Circeis* mean size between SST and LST (*p* = .238, Student's *t*‐test), large shrimp left *Circeis* survivors significantly larger than small shrimp (*p* = .049, Student's *t*‐test), which means higher escape size in LST. Large shrimp significantly decreased the mean colony size in *Juxtacribrilina*. In *Celleporella*, small shrimp decreased the mean colony size, while large shrimp increased it. Large shrimp eradicated the prevalence of the smallest colonies in *Celleporella* so that the dominance shifted to a larger size class (Figure 5).

**TABLE 6:** Mean individual sizes of dominant prey species before and after *Spirontocaris phippsii* predatory shrimp exposure in the laboratory experiment.

Shrimp size	Survivors in a trial	*Circeis armoricana*				*Juxtacribrilina annulata*				*Celleporella hyalina*				*Electra pilosa*		
		*N*	Aperture diameter, mm			*N*	Number of zooids			*N*	Number of zooids			*N*	Number of zooids	
			Before		After		Before		After		Before		After		Before	After
Small	With survivors	35	**0.176 ± 0.002**	<	**0.190 ± 0.003**	32	3.9 ± 0.4		3.4 ± 0.4	32	**32.8 ± 3.4**	>	**27.2 ± 3.2**	18	6.8 ± 1.1	6.4 ± 1.8
	W/o survivors	0	**–**		**–**	3	3.8 ± 0.5		**–**	2	10.5 ± 0.5		**–**	15	5.2 ± 0.8	**–**
Large	With survivors	18	**0.181 ± 0.004**	<	**0.203 ± 0.006**	9	**4.7 ± 0.8**	>	**2.5 ± 0.6**	9	**30.6 ± 3.3**	<	**39.5 ± 5.2**	2	7.9 ± 3.1	3.4 ± 1.4
	W/o survivors	0	**–**		**–**	9	3.8 ± 0.6		**–**	8	27.6 ± 12.1		**–**	15	6.2 ± 0.9	**–**

*Note*: Significant differences (exact Wilcoxon–Pratt signed‐rank test, *p* < .05) in the trials with survivors highlighted in bold, “<” – mean size increased, “>” – mean size decreased.

**FIGURE 6:**
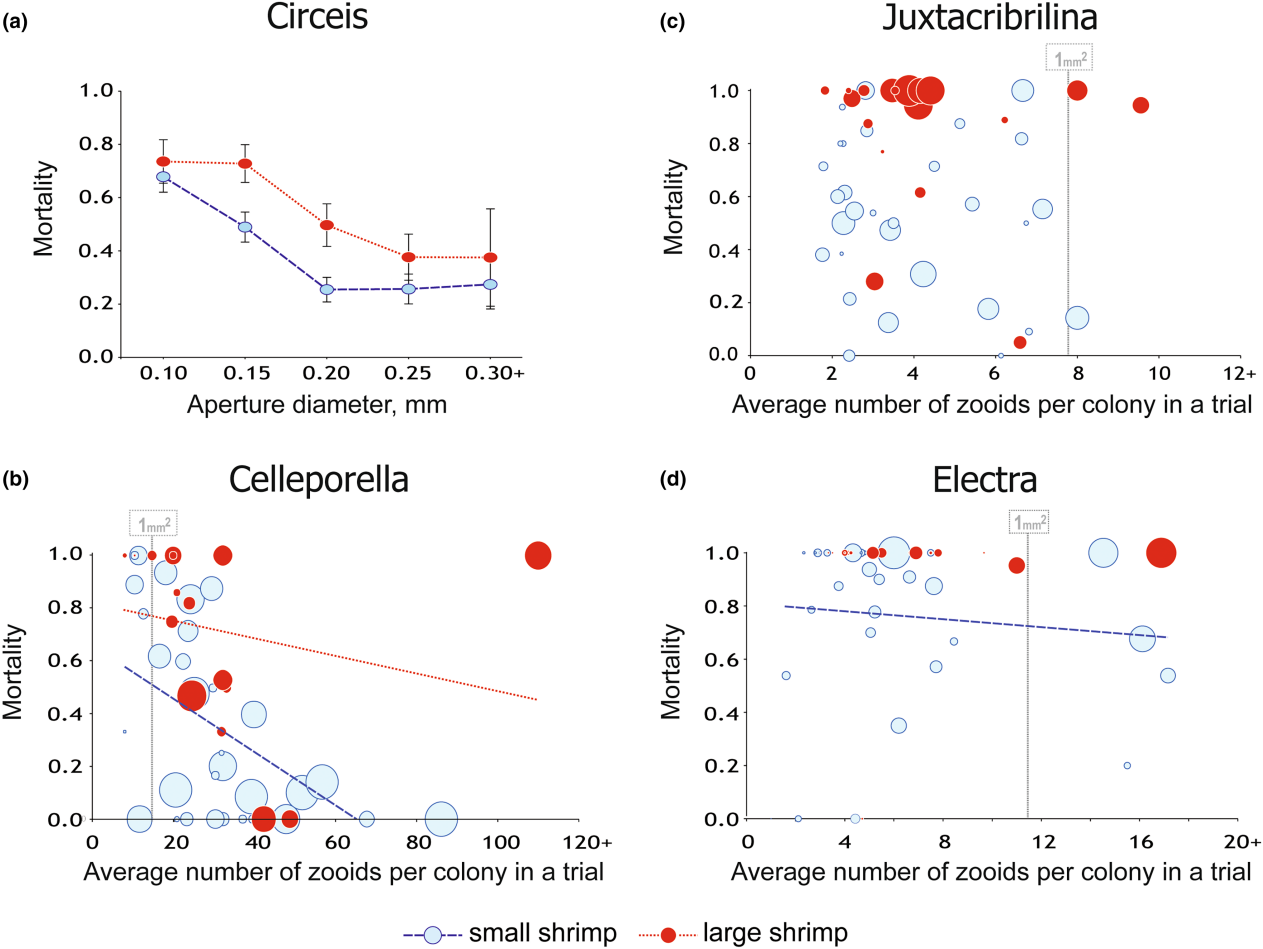
Relationships between the mortality of dominant epibenthic species on *Phycodrys rubens* and their individual size (for *Circeis armoricana*) or the initial mean colony size in a trial (for bryozoans), their relative percent covers, and *Spirontocaris phippsii* predatory shrimp size in the laboratory experiments. In bryozoans, each circle denotes a trial, and circle area shows relative percent cover of the corresponding species in a trial. Area threshold of 1 mm^2^ covered by a colony marked for bryozoans. Trend lines indicate the relationship where the effect of mean colony size on mortality is significant (see Table 6).

Table 7 and Figure 6 summarize the effects of initial prey size, its relative and absolute abundance, and predator size on the mortality of dominant prey species. *Circeis* individual size had strong negative effect on its mortality, whereas shrimp size increased it. In bryozoans, partial predation only allowed an indirect assessment of size–mortality relationship based on mean initial colony size in a trial (hereafter “MIS”), and the response was species‐specific. *Celleporella* mortality decreased with higher MIS along with the increase in the probability of total survival. The probability of *Celleporella* total elimination increased with shrimp size. *Electra* also showed a mortality decrease with higher MIS, but only in SST. In LST, *Electra* was almost totally eliminated, regardless of the demographic traits. In *Juxtacribrilina*, neither MIS nor initial relative or absolute abundance affected mortality. The probability of total elimination, however, was higher in LST. While the initial number of colonies positively correlated with the survival rate in *Celleporella* and *Electra*, the initial share of substrate area covered (relative abundance) had a negative effect on survival in *Circeis*, *Celleporella*, and *Electra* (in SST). Neither absolute nor relative initial abundance had any effect on *Juxtacribrilina* mortality. The relatively predation‐safe *Circeis* individuals with an aperture size of 0.25 mm and larger were initially present in 94% of the trials. In contrast, *Celleporella* colonies larger than 50 zooids in size were 60% frequent, and *Celleporella*'s MIS exceeded 30 zooids only in 42% of the trials.

**TABLE 7:** Effects of *Spirontocaris phippsii* shrimp size, individual prey size (for *Circeis*) or initial mean prey colony size in a trial (for *Juxtacribrilina*, *Celleporella*, and *Electra*), initial prey number, and relative cover on prey mortality in the laboratory experiment: zero‐and‐one‐inflated (logit link) beta‐regression, mean (logit link), and variance (log link) modeled.

Prey species	Source of variation	Mean model (Mu)		Zeros model (Nu)		Ones model (Tau)	
		Estimate	*p*	Estimate	*p*	Estimate	*p*
*Circeis armoricana*	Intercept	1.15 ± 0.27	<.001	−3.52 ± 0.90	<.001	0.98 ± 0.82	.235
	Shrimp size [large, small]	**0.50 ± 0.14**	**<.001**	−0.36 ± 0.47	.446	**1.24 ± 0.51**	**.017**
	Prey size	**−8.76 ± 1.29**	**<.001**	**17.45 ± 3.50**	**<.001**	**−7.77 ± 3.36**	**.022**
	Initial prey number	−0.01 ± 0.01	.266	**−0.09 ± 0.03**	**<.001**	**−0.19 ± 0.04**	**<.001**
	Initial prey relative cover	**1.22 ± 0.47**	**.010**	−0.30 ± 1.58	.849	0.88 ± 1.73	.611
*Juxtacribrilina annulata*	Intercept	1.64 ± 0.64	.023	57 ± 4939	.991	1.58 ± 1.44	.290
	Shrimp size [large, small]	0.70 ± 0.47	.162	21 ± 4408	.996	**1.80 ± 0.77**	**.033**
	Initial mean prey colony size	−0.10 ± 0.08	.217	4 ± 394	.991	−0.24 ± 0.21	.270
	Initial prey number	0.01 ± 0.02	.498	−9 ± 221	.967	−0.08 ± 0.06	.216
	Initial prey relative cover	−3.60 ± 2.06	.102	236 ± 10,027	.982	4.25 ± 4.07	.314
*Celleporella hyalina*	Intercept	3.60 ± 0.19	<.001	−2.57 ± 1.42	.103	7.70 ± 2.01	.004
	Shrimp size [large, small]	**1.91 ± 0.12**	**<.001**	−1.01 ± 1.00	.338	**7.16 ± 2.05**	**.006**
	Initial mean prey colony size	**−0.11 ± 0.01**	**<.001**	**0.09 ± 0.04**	**.043**	−0.14 ± 0.08	.089
	Initial prey number	**−0.20 ± 0.01**	**<.001**	0.05 ± 0.11	.650	−0.34 ± 0.32	.313
	Initial prey relative cover	**7.40 ± 0.68**	**<.001**	−5.92 ± 3.85	.157	2.90 ± 12.93	.827
*Electra pilosa* (small shrimp trials only, no variances model)	Intercept	0.45 ± 0.25	.155	50 ± 463	.920	3.56 ± 1.40	.070
	Initial mean prey colony size	**−0.18 ± 0.02**	**.002**	−25 ± 352	.947	−0.15 ± 0.17	.443
	Initial prey number	**−0.16 ± 0.02**	**.003**	−7 ± 125	.960	−0.55 ± 0.23	.079
	Initial prey relative cover	**26.38 ± 2.72**	**.001**	827 ± 14,297	.957	20.77 ± 10.93	.137

*Note*: Predator size used as a sole predictor in variances model. Since *Electra* was almost totally eliminated in large shrimp trials, the model for *Electra* was based on small shrimp trials, and variances were not modeled. Random blocking effects for means model were Shrimp ID for all the species and also Trial ID nested in Shrimp ID in *Circeis*. Significant terms (*p* < .05) highlighted in bold.

Individual tubeworms were mostly larger than bryozoan zooids and smaller than their colonies. *Juxtacribrilina* had the largest zooids, similar in size to the smallest tubeworms. *Electra* had smaller zooids, and *Celleporella* had the smallest ones. On the other hand, *Celleporella* initially had, on average, larger colonies (~2 mm^2^) than more numerous *Juxtacribrilina* and *Electra* (~0.5 mm^2^). Importantly, even the largest (~6 mm^2^) *Celeporella* colonies were several times less than a mean daily ration of large shrimp. There was, therefore, no direct limitation on the size of a colony a shrimp could consume in a 24‐h trial.

In SST, but not in LST, the largest bryozoan colonies in a trial generally lost a smaller fraction of the substrate area covered compared to the rest of the population, though they were often partially injured (Table 8). The result for *Juxtacribrilina*, though, is biased toward the cases where the largest colony was either untouched or entirely consumed, since its size structure rarely allowed tracing individual partially damaged colonies.

**TABLE 8:** Mean cover loss by the largest bryozoan colony in a trial compared to the mean cover loss by all the colonies in the same trial after exposure to *Spirontocaris phippsii* predatory shrimp in the laboratory experiment.

	*Celleporella hyalina*		*Electra pilosa*		*Juxtacribrilina annulata* ^a^	
	SST	LST	SST	LST	SST	LST
Total cover loss (%)	31 ± 6	67 ± 10	78 ± 7	94 ± 6	53 ± 9	90 ± 10
Largest colony cover loss (%)	21 ± 6	59 ± 12	68 ± 10	94 ± 6	37 ± 11	90 ± 9
*N*	27	15	23	16	20	10
*Z*	−3.25	−1.43	−2.44	n.a.	−3.24	−1
*p*	**<.001**	.141	**.031**	n.a.	**<.001**	>.999

*Note*: Wilcoxon–Pratt signed‐rank test. Calculations based only on the trials where the fate of the largest colony could be traced from the size structure. Significant terms (*p* < .05) highlighted in bold.

^a^ The size structure of *Juxtacribrilina* rarely allowed tracing individual partially damaged colonies; thus, the result for *Juxtacribrilina* could be biased toward the cases where the largest colony was either untouched or entirely consumed.

## 4 DISCUSSION

Our experiments prove that large and small predator individuals differently affect prey community composition, particularly the proportion of unitary and modular organisms. Consistent with our prediction, the ability to outgrow predation pressure favored the survival of unitary prey over modular. The unitary tubeworms' vulnerability decreased with their size; therefore, the largest individuals were nearly immune to shrimp predation. Modular bryozoans' response, however, was species and size specific. The role of partial predation in improving modular prey survival was subordinate, since the difference between mortality and reduction of substrate area covered was small. This disagrees with a general consideration that consumer control of modular organisms primarily affects module survival, with percent covers and numbers of modules being more stable than numbers of individual clones (Hughes & Jackson, 1985; Tuomi & Vuorisalo, 1989). However, the pattern we observed is probably a habitat‐specific one: unlike other habitats (Rubin, 1987; Walters, 1992), young *Phycodrys* parts do not provide refuges, which could partially shield bryozoan colonies from consumer control and thus promote partial predation as a defense mechanism.

Using natural epibiosis in our experiments results in certain limitations caused by initial species‐specific differences in size structures. First, it is possible that the higher initial frequency of smaller bryozoan colonies contributed to their lower survival due to higher predator encounter probability. This presumably did not have any significant effect, since *Juxtacribrilina* was consumed regardless of mean colony size. Second, our current setup had no power to clearly separate the effects of prey species and colony size on bryozoans' survival rate. *Celleporella* showed relatively high survival rates in the trials, with a mean colony size of at least 25–30 zooids (2 mm^2^), while most of the *Juxtacribrilina* and *Electra* colonies were within 1 mm^2^ before treatments. On less ephemeral substrates, both *Electra* and *Juxtacribrilina* commonly sustain colony sizes reaching hundreds or thousands of zooids (Chava et al., 2019; Nekliudova, Shunkina, et al., 2019; Shevchenko et al., 2020), possibly having higher predation survival rates. Also, the brief starvation imposed on shrimp before the trials could potentially reduce their feeding selectivity (Perry, 1987). Given that the sequence number of a replicate trial neither affected the proportion of total epibenthic cover cleared by shrimp nor the species diversity of the prey consumed, we consider this bias as negligible.

Variation in mechanical defenses of the prey taxa against predation best explains the observed differential survival patterns. Vulnerability to predators strongly depends on defensive structures' effectiveness, and appears much higher in soft than calcareous epibionts; the proportion of soft sessile organisms increases in the absence of consumer control regardless of their unitary or modular organization (Dias et al., 2020; Osman et al., 1992; Vieira et al., 2012). Consequently, the contrast between the defensive strategies of unitary and modular organisms likely defines consumer control of community structure only when they share similar armor, that is, are both either soft or calcareous. Here, the primary defensive structure in dominant taxa (which are all calcareous) is their body wall. Its thickness and, consequently, strength are a function of body size in unitary organisms (e.g., tubeworms, barnacles, or bivalves) and module size in modular ones (e.g., bryozoans). The larger the predator (e.g., shrimp) the thicker the body wall it can crash. Tubeworms grow much larger than any bryozoan zooid, have a thickest body wall, and consistently show the highest resistance to predation pressure. All the bryozoans are less protected because of the zooid size limit. *Celleporella* has the smallest zooids (and consistently the lowest survival of earliest recruits; see Figure 5). The interspecific difference in colony organization possibly explains the superior survival of larger *Celleporella* colonies compared to other bryozoans: unlike *Juxtacribrilina* and *Electra*, which develop single‐layer encrusting colonies, *Celleporella* emerges generative zooids to a second “frontal” layer, spreading from the center of a colony, so that only the peripheral part is thin (Ostrovsky, 1998). *Electra* has large spines, potentially providing a mechanical defense from predators (Lidgard, 2008), which leaves the least protected *Juxtacribrilina* a preferable or most vulnerable prey consumed regardless of its relative abundance or size.

Larger module size is considered a competitive advantage, defining an evolutionary trend in bryozoans (Liow et al., 2019). Colony thickness coming with age is also known to improve their competitive ability (Buss, 1980). There is, however, no established relationship between these traits and resistance to consumers. Our data suggest that while module size may determine the vulnerability of early recruits to predation, the ability to resist predation pressure in larger colonies is more related to colony organization and defensive structures.

The observed higher vulnerability of modular organisms to predation is probably balanced by their complementary advantage in competition (Jackson, 1977; Keough, 1984). Consistently, *Circeis*, *Celleporella*, and *Juxtacribrilina* seem to comprise the inverse competitive and predation‐resistance hierarchies. *Circeis*, like other serpulid worms (Keough, 1984), is regularly overgrown by many bryozoans, including specifically *Juxtacribrilina* (see fig. 4 in Meyer et al., 2017). *Juxtacribrilina annulata* is a rather strong competitor (Barnes & Kukliński, 2003), and a closely related *Cribrilina cryptooecium* outcompetes *Celleporella* and *Electra* in overgrowth interactions (Turner & Todd, 1994). *Celleporella* overgrows *Electra* (Cancino & Hughes, 1988). Given that competitive strength in modular organisms is also often a function of colony size (Buss, 1980), the net relative performance of unitary and modular taxa would be a complex product of their and consumer abundance and size structure combined with substrate availability in a particular microhabitat.

Epibiosis is commonly regulated by direct and indirect effects of facilitation by a host foundation species (“basibiont”), which may include mediation of top‐down effects (Stelling‐Wood et al., 2023). Together with substrate, basibionts commonly provide their epibionts with refuges from predation (Walters, 1992). Foundation species can shape dependent assemblages by variation (e.g., ontogenetic) of individual properties (Taylor & Burns, 2015). *Phycodrys rubens* in the White Sea supports different species composition on its young blades (where competition for space is negligible) depending on the size of the plant, with a higher proportion of *Circeis* on larger hosts (Chava et al., 2019). This pattern suggests larger plants may encourage higher predation pressure. Birds, for instance, would heavier attack caterpillars on mature trees compared to saplings, making top‐down control the hidden mechanism underlying ontogenetic shift in facilitation (Zvereva et al., 2020). Most substrate area provided by *Phycodrys* consists of large young blades (used in our experiments), which start growing in spring and are loosely covered by epibionts (about 8% total covers in September, see Chava et al., 2019). *Phycodrys* spans up to 4 years (Schoschina, 1996), and its blades partially degrade in winter, making older plant parts smaller and much more space limited (up to 40% total covers) due to the accumulation of winter‐surviving epibionts (Chava et al., 2019). Consequently, ontogenetic changes and individual size variation in a foundation species may switch the balance of predation and competition in its epibiosis.

The impact predator size can have on the proportion of unitary and modular organisms makes the spatio‐temporal variation in predator size and age structure an important driver of epibiotic community structure. In the case of *Spirontocaris*, with a life span of up to 5 years (Węsławski, 1987), large‐scale migrations (Allen, 1962; Pike, 1954) and, possibly, higher‐order size‐selective predators (Jónsdóttir, 2017; Mehl, 1991) may shape its local abundance and population structure. Climatic variables also have strong location‐specific influence on shrimp recruitment and hence drive their size structure (Beukema, 1992; Henderson et al., 2006). The traces of interannual variation in abiotic conditions can accumulate in long‐living keystone predators' population structure and cause indirect effects on dependent assemblages of short‐living unitary and modular sessile organisms. Variability in reproductive output of prey between species, year, substrates, and short‐lived versus overwintering generations (Nekliudova, Schwaha, et al., 2019; Nekliudova, Shunkina, et al., 2019; Shevchenko et al., 2020) possibly provides additional long‐term feedbacks.

Generalist predators supposedly stabilize assemblages at lower trophic levels by absorbing the species‐specific abundance surges (Post et al., 2000). Ontogenetic shifts in their diet are, however, ubiquitous (Stallings et al., 2023), which should imprint their demography in top‐down, controlled community structure. Recently, even minor ontogenetic diet shifts in predators have been theoretically predicted to cause the shaping of prey community by predator population structure via creating emergent competition–predation trade‐offs between competing prey species (Wollrab et al., 2013). Treating a prey population not as a homogeneous entity but instead considering prey size classes as different functional groups is also believed critical to reliably predict community dynamics (Rudolf, 2008). Further research should expand the range of systems to investigate where the relative performances of unitary and modular sessile organisms are potentially affected by predator population structure. Similar to recent studies of competition (Lasky et al., 2015), assessing the role of consumer control should wider encompass ontogenetic shifts in the functional traits of predators and prey to predict the outcome of their interactions.

## AUTHOR CONTRIBUTIONS

**Alexandra Chava:** Conceptualization (equal); data curation (supporting); investigation (lead); methodology (equal); writing – original draft (supporting); writing – review and editing (equal). **Anna Artemieva:** Conceptualization (equal); data curation (supporting); investigation (supporting); methodology (equal); writing – original draft (supporting); writing – review and editing (equal). **Eugeniy Yakovis:** Conceptualization (equal); data curation (lead); funding acquisition (lead); investigation (supporting); methodology (equal); project administration (lead); writing – original draft (lead); writing – review and editing (equal).

## CONFLICT OF INTEREST STATEMENT

The authors declare no conflict of interest.

## Supporting information


Appendix S1

## Data Availability

Data are provided as a Supporting Information file.
